# Hypothalamic Temperature of Rats Subjected to Treadmill Running in a Cold Environment

**DOI:** 10.1371/journal.pone.0111501

**Published:** 2014-11-03

**Authors:** Cletiana Gonçalves Fonseca, Washington Pires, Milene Rodrigues Malheiros Lima, Juliana Bohnen Guimarães, Nilo Resende Viana Lima, Samuel Penna Wanner

**Affiliations:** 1 Exercise Physiology Laboratory, School of Physical Education, Physiotherapy and Occupational Therapy, Universidade Federal de Minas Gerais, Belo Horizonte (MG), Brazil; 2 Instituto Superior de Educação Anísio Teixeira, Universidade Estadual de Minas Gerais, Ibirité (MG), Brazil; Inserm U837, France

## Abstract

Different strategies for cooling the body prior to or during physical exercise have been shown to improve prolonged performance. Because of ethical and methodological issues, no studies conducted in humans have evaluated the changes in brain temperature promoted by cooling strategies. Therefore, our first aim sought to measure the hypothalamic temperature (T_hyp_) of rats subjected to treadmill running in a cold environment. Moreover, evidence suggests that T_hyp_ and abdominal temperature (T_abd_) are regulated by different physiological mechanisms. Thus, this study also investigated the dynamics of exercise-induced changes in T_hyp_ and T_abd_ at two ambient temperatures: 25°C (temperate environment) and 12°C (cold). Adult male Wistar rats were used in these experiments. The rats were implanted with a guide cannula in the hypothalamus and a temperature sensor in the abdominal cavity. After recovery from this surgery, the rats were familiarized with running on a treadmill and were then subjected to the two experimental trials: constant-speed running (20 m/min) at 12°C and 25°C. Both T_hyp_ and T_abd_ increased during exercise at 25°C. In contrast, T_hyp_ and T_abd_ remained unchanged during fatiguing exercise at 12°C. The temperature differential (i.e., T_hyp_ - T_abd_) increased during the initial min of running at 25°C and thereafter decreased toward pre-exercise values. Interestingly, external cooling prevented this early increase in the temperature differential from the 2^nd^ to the 8^th^ min of running. In addition, the time until volitional fatigue was higher during the constant exercise at 12°C compared with 25°C. Together, our results indicate that T_hyp_ and T_abd_ are regulated by different mechanisms in running rats and that external cooling affected the relationship between both temperature indexes observed during exercise without environmental thermal stress. Our data also suggest that attenuated hypothalamic hyperthermia may contribute to improved performance in cold environments.

## Introduction

The core body temperature (T_core_) of homeothermic animals is maintained within narrow limits over a wide range of ambient temperatures. T_core_ is tightly regulated through adjustments in the rates of heat production and heat dissipation, which are in turn controlled by the activation or inhibition of autonomic and behavioral thermoeffectors [1], [2]. In rats, T_core_ can be measured at different body sites, including the abdomen, rectum and brain. Some authors have suggested that brain temperature is more important than the other indexes of T_core_ for controlling thermoeffector recruitment [3], [4], although no consensus exists regarding this issue [5].

The level at which brain temperature is regulated depends primarily on the heat produced by brain tissue metabolism. In humans, the brain represents only ∼2% of the body mass but accounts for ∼20% of the whole body oxygen consumption under resting conditions [6]. To maintain brain homeostasis, heat must be dissipated from the brain to the body and then to the external environment. The primary route for the dissipation of brain-generated metabolic heat is the cerebral circulation, as evidenced by the fact that the temperature of jugular venous blood is always higher than the temperature of arterial blood (aortic) during exercise [7]. Experimental evidence indicates that cerebral blood flow increases during light to moderate-intensity exercise [8], [9].

In the theoretical model proposed by Cheung and Sleivert [10] to explain the association between hyperthermia and exercise fatigue, brain temperature was indicated as one of the factors regulating prolonged physical performance. This theoretical model suggests that the exercise-induced increase in brain temperature decreases alertness [11] and increases the perception of effort [12], leading to volitional fatigue. Because prolonged physical performance is reduced at high brain temperatures [13], [14], [15], the effectiveness of several strategies for cooling the body (and likely the brain) prior to or during exercise has been investigated. Generally, these experimental procedures are effective at reducing thermoregulatory strain and improving physical performance [16]. However, because of ethical and methodological issues, no studies conducted in humans have evaluated the changes in brain temperature promoted by cooling strategies. Therefore, our first aim sought to measure the hypothalamic temperature (T_hyp_) of rats subjected to treadmill running in a cold environment. Exposure to an ambient temperature of 12°C was used as a strategy for externally cooling the rat’s body.

The temperatures measured in specific body compartments are not homogeneous and do not respond similarly (particularly regarding their time courses) to feeding [17] and several arousing stimuli, such as light, sound, and tail-pinch [18]. Although brain and abdominal temperatures have been simultaneously recorded in running rats [13], [14], the dynamics of the exercise-induced increases in both T_core_ indexes have not been compared throughout the exercise period. During treadmill running, blood flow is shunted from the viscera to the active skeletal muscles, heart, brain and skin [19], [20], [21]. As a consequence of this cardiovascular drift, visceral blood flow may be greatly reduced [19], [22], which may ultimately change T_abd_. In the light of previous evidence, we hypothesized that T_hyp_ and T_abd_ may follow different patterns in response to physical exertion. Thus, the present study also aimed to investigate the relationship between T_hyp_ and T_abd_ during physical exercise at two ambient temperatures.

## Experimental Procedures

### Animals

Twenty-two adult male Wistar rats weighing 250–365 g were used in all experiments. The animals were housed in individual cages under controlled light (05∶00–19∶00 h) and temperature (24.5±1.1°C) conditions, with water and rat chow provided *ad libitum*. All experimental procedures were approved by the Ethics Committee of the Universidade Federal de Minas Gerais for the Care and Use of Laboratory Animals (protocol 278/10) and were performed in accordance with the policies described in the Committee’s Guiding Principles Manual.

### Experimental protocols

Two sets of experiments were conducted to achieve the goals of the present study. The first set consisted of control experiments and was conducted to determine the exercise capacity of the rats (inferred from the maximum treadmill speed attained during incremental-speed running) at ambient temperatures of 12°C and 25°C (cold and temperate environments, respectively). The incremental exercise-induced changes in abdominal temperature were also investigated at these two ambient temperatures. Initially, the rats were implanted with a temperature sensor in the abdominal cavity. After recovery from this surgical procedure, the animals were familiarized with running on a treadmill. The rats were then subjected to the experimental trials (incremental exercises at 12°C and 25°C) with an interval of 48 h between the trials. The ambient temperature was controlled by an environmental chamber (Russells Technical Products, WMD 1150-5, MI, USA). After the last experimental trial, the animals were euthanized with a lethal dose of anesthetic.

The second set of experiments sought to measure the T_hyp_ of rats subjected to treadmill running in a cold environment. These experiments also addressed the dynamics of the exercise-induced changes in T_hyp_ and T_abd_ at two ambient temperatures, 25°C and 12°C. The animals from this second set of experiments were subjected to the same procedures as the animals from the first set, except that they were subjected to constant-speed exercises rather than incremental-speed exercises and they were also implanted with a hypothalamic guide cannula (under the same anesthesia used for the implantation of the abdominal sensor). After the last experimental trial, the animals were euthanized, and their brains were removed for identification of the thermistor tip locations.

### Implantation of an abdominal temperature sensor and of a hypothalamic guide cannula

The animals were anesthetized with intraperitoneal (i.p.) ketamine and xylazine (90 and 10.5 mg/kg of body mass, respectively). Immediately prior to the surgery, the animals were treated with a prophylactic dose of intramuscular antibiotics (48,000 IU of veterinary pentabiotic for small animals, 0.1 mL applied in each hind paw) and subcutaneous analgesic medication (flunixin meglumine, 2 mg/kg). Once the rats were anesthetized, a temperature sensor (G2 E-Mitter, Mini-Mitter Respironics, OR, USA) was implanted in the abdominal cavity using a previously described technique [23].

The animals used in the second set of experiments had a guide cannula implanted into their right ventromedial hypothalamus (VMH) to allow the insertion of a thermistor for measuring the hypothalamic temperature. The implantation of the guide cannula preceded the implantation of the abdominal sensor. The animals were fixed to a stereotaxic apparatus (Insight Equipamentos, SP, Brazil), and a sterile stainless steel cannula (20.0 mm long, 0.8 mm OD, 21 gauge) was stereotaxically implanted into the hypothalamus such that the tip of the cannula was aimed at the dorsal portion of the VMH, according to the following coordinates: AP, 2.5 mm posterior to bregma; ML, 0.6 mm from the midline; and DV, 8.5 mm deep from the dura mater [24]. The cannula was anchored firmly to the screws with acrylic cement. A stainless steel obturator (0.1 mm diameter) was placed inside the guide cannula to prevent obstruction of the lumen. The animals were allowed to recover from these surgical procedures for at least 3 days; this recovery period was sufficiently long for the rats to regain their pre-operative body mass.

We tried to measure the VMH temperature as an index of T_hyp_ because previous evidence indicated that the VMH modulates physical performance [25] and is involved in the neural pathways that mediate exercise-induced metabolic [26], [27], [28], thermoregulatory [29], and cardiovascular responses [30].

### Familiarization with the treadmill exercise

The rats were encouraged with light electrical stimulation (0.5 mA) to exercise on a motor-driven treadmill for small animals (Columbus Instruments, OH, USA). The familiarization protocol consisted of the rats running at a constant speed of 18 m/min for 5 min for 5 consecutive days prior to the experiments [25], [29]. The exercise familiarization sessions were performed at a dry ambient temperature of 25°C and a relative humidity of 50%. The treadmill inclination was always set at 5%, and an electrical fan, which generated an airflow rate of 2.0–2.5 m/min, was positioned immediately in front of the treadmill belt and used throughout the exercise during all of the familiarization sessions and experimental trials. The purpose of these familiarization sessions was to teach the animals the direction in which they should run and to minimize their exposure to the electrical stimuli during the experimental trials [25].

### Incremental-speed exercises

The incremental exercises were initiated with a treadmill speed of 10 m/min, which was increased by 1 m/min every 2 min until the rats were fatigued [23], [28]. Volitional fatigue was defined as the moment when the animal could no longer keep pace with the treadmill while undergoing light electrical stimulation for 10 s [23], [31]. The experiments were performed between 13∶00 and 18∶00 h, and the order of the trials was randomized and balanced.

### Constant-speed exercises

These exercises were performed at a constant speed of 20 m/min until volitional fatigue. We selected this exercise intensity based on early findings from Guimaraes *et al.* [25], who reported that the T_abd_ of rats did not change throughout a fatiguing treadmill run at a constant speed of 20 m/min at 12°C. The control trials were conducted at an ambient temperature of 25°C because treadmill speeds ranging from 18 to 24 m/min previously produced marked increases in T_abd_ and brain temperature under these conditions [15], [23].

### Measurements

T_hyp_ was continuously measured using a thermistor (NTC Catheter Sensor, Measurements Specialties, VA, USA) that was inserted into the hypothalamus prior to each experimental trial. The thermistor (183 cm length and 38 AWG) is flexible, allows for fast, accurate measurements and was inserted 0.2 mm past the tip of the guide cannula. The resistance values were recorded by a digital multimeter (Fluke, WA, USA) and then converted into temperature values using linear regression.

T_abd_ was recorded every 15 s by telemetry. The radio waves emitted by the abdominal sensor were captured by a receiving plate (ER-4000 energizer/receiver, Mini-Mitter Respironics) positioned next to the treadmill. The radio wave frequencies were converted into temperature values by software (Vital View), and the data were stored online. The ambient temperature inside the treadmill was measured every min using a thermocouple (Yellow Spring Instruments, YSI; OH, USA) positioned on top of the acrylic chamber that contained the treadmill belt.

### Calculations

The rate of increase in the T_hyp_ and T_abd_ was calculated using the following equation: RIT (°C/min) = [T_fatigue_–T_initial_]/TET; where RIT is the rate of increase in temperature, T_fatigue_ (°C) is the temperature at volitional fatigue, T_initial_ (°C) is the temperature at the beginning of exercise, and TET (min) is the total exercise time. The hypothalamic-abdominal temperature differentials were calculated by subtracting T_abd_ from T_hyp_. This temperature differential can help determine whether T_hyp_ changes arise from central or peripheral sources [18], [32].

In the first set of experiments, the maximum running speed attained during the incremental exercises was considered the physical performance index. The maximum speed (S_max_) was calculated by modifying the equation proposed by Kuipers *et al.* [33] for calculating the maximal power output: *S_max_ = S1+(S2*×*t/120)*, where *S1* is the speed reached in the last completed stage, *S2* is the increment in the treadmill speed in each stage, and *t* is the time spent (in seconds) in the uncompleted stage. In the second set of experiments, the total exercise time until volitional fatigue was considered the performance index.

### Euthanasia and histological verification

After the last experimental trial, the animals from the first set of experiments (not implanted with a hypothalamic guide cannula) were euthanized with a lethal dose of ketamine and xylazine (270 and 31.5 mg/kg i.p., respectively).

In contrast, the animals from the second protocol were deeply anesthetized with ketamine and xylazine (135 and 16 mg/kg i.p., respectively). The ascending aorta was cannulated, and an incision was made in the right atrium. The animal was perfused with 100 mL of 0.9% saline containing heparin (10 U/mL), followed by perfusion with 300 mL of 4% formaldehyde. The brain was removed and post-fixed in 4% formaldehyde at 4°C. Three days prior to brain slicing, the tissue was transferred to a solution containing 30% sucrose diluted in 4% formaldehyde. The brain was cut into 50 µm slices at −20°C on a cryostat (Microm HM505N, NSW, Australia). The slices were fixed on gelatinized slides and stained with a 0.13% cresyl violet solution. The position of the thermistor tips was determined by comparing the lesioned areas present in the slides with coronal drawings in the Paxinos and Watson atlas [24].

### Statistical analysis

Data are expressed as the mean ± SEM. The T_hyp_ and T_abd_ curves were compared between the experimental trials (i.e., 25°C and 12°C) and among time points using a repeated measures two-way analysis of variance, followed by the Tukey *post hoc* test. The total exercise time, the maximum speed attained during the incremental exercises and the rates of increase in T_hyp_ and T_abd_ were compared between trials using paired Student’s *t* tests. The effect size (Cohnen’s *d* for two dependent means) was calculated by subtracting the mean of the performance index at 12°C from the mean of the same index at 25°C and then dividing the result by the pooled standard deviation for the data. Moreover, the plots of the number of running animals vs. total exercise time were compared using the logrank test. The association between physical performance and the rate of increase in T_hyp_ or T_abd_ was assessed using the Pearson correlation coefficient. The level of significance was set at *P*<0.05.

## Results

### Incremental-speed exercise

**Figure 1:**
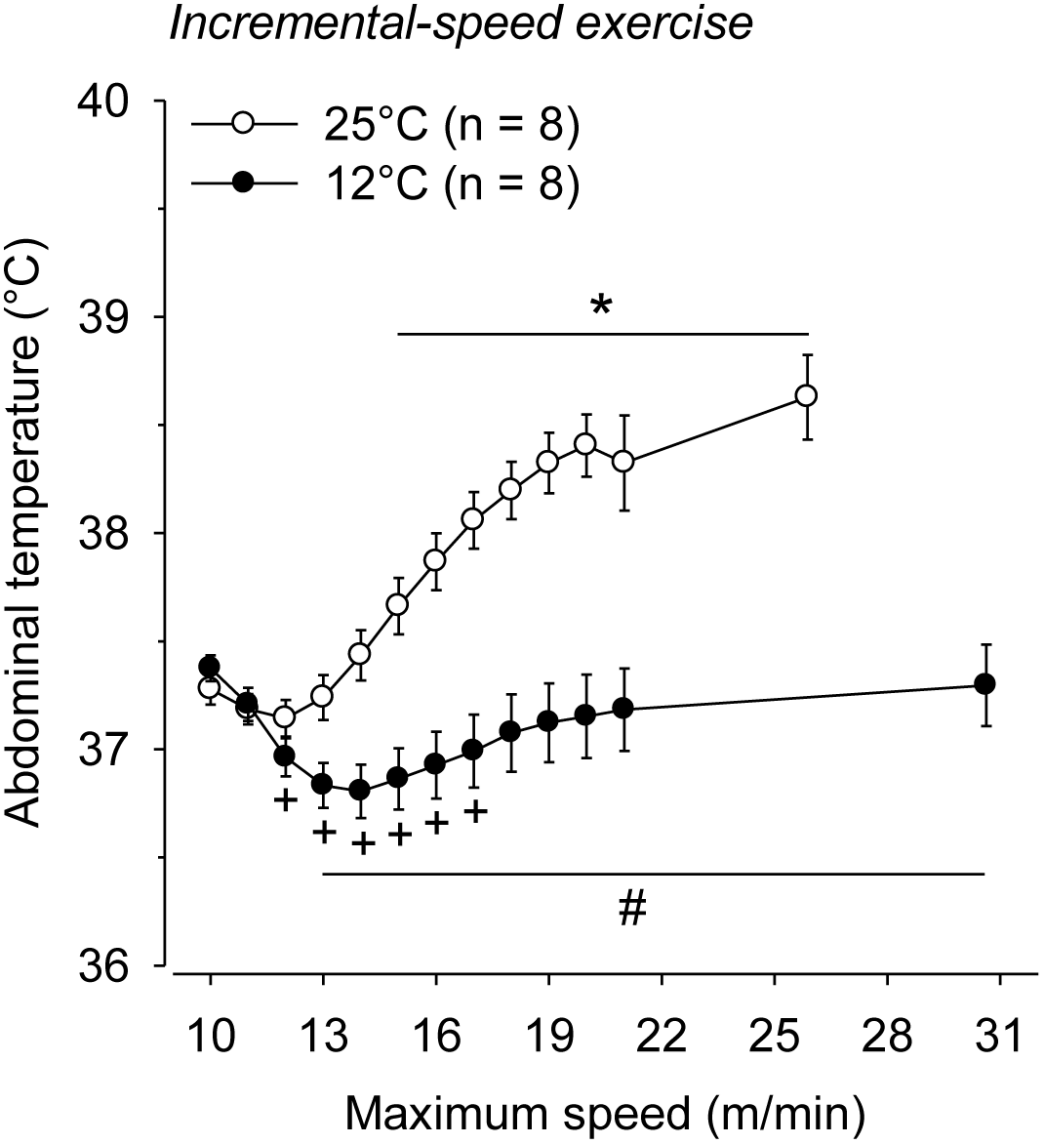
Abdominal temperature during incremental-speed exercises that were performed until volitional fatigue at ambient temperatures of 25°C and 12°C. *P<0.05 compared with the beginning of the exercise at 25°C; +P<0.05 compared with the beginning of the exercise at 12°C; #*P*<0.05 compared with the experimental trial at 25°C.

At 25°C, the rats ran for 33.2±3.6 min and attained an S_max_ of 25.6±1.8 m/min, indicating that they completed the 16^th^ stage of the incremental exercise. The exercise capacity was markedly increased in the cold condition. The total exercise time and S_max_ attained during the treadmill running were 28.9% and 18.8% higher, respectively, when the rats performed the exercise at 12°C (Table 1). During incremental running at 25°C, T_abd_ increased from the 6^th^ stage of exercise, continued to increase until the 12^th^ stage and then reached a plateau that was sustained until volitional fatigue (38.64±0.19°C; *P*<0.001). In contrast, at 12°C, T_abd_ slightly decreased from the 3^rd^ until the 8^th^ stage (36.80±0.12 at 14 m/min vs. 37.37±0.06°C at the beginning of exercise; *P*<0.01). Thereafter, T_abd_ increased gradually toward resting values such that the values recorded at volitional fatigue were not different from those recorded prior to the exercise (37.31±0.18°C vs. 37.37±0.06°C, *P* = 0.66; Figure 1).

**Table 1:** Indexes of physical performance in rats subjected to incremental-speed exercises at ambient temperatures of 12 and 25°C.

Performance index	25°C	12°C	Increase(%)	Statistics*P* value	Effect size
Running time (min)	33.2±3.6	42.9±1.5	28.9	0.015	1.13
Maximum speed (m/min)	25.6±1.8	30.4±0.8	18.8	0.015	1.13
Distance traveled (m)	616±101	864±51	40.4	0.021	1.05

### Constant-speed exercise

**Figure 2:**
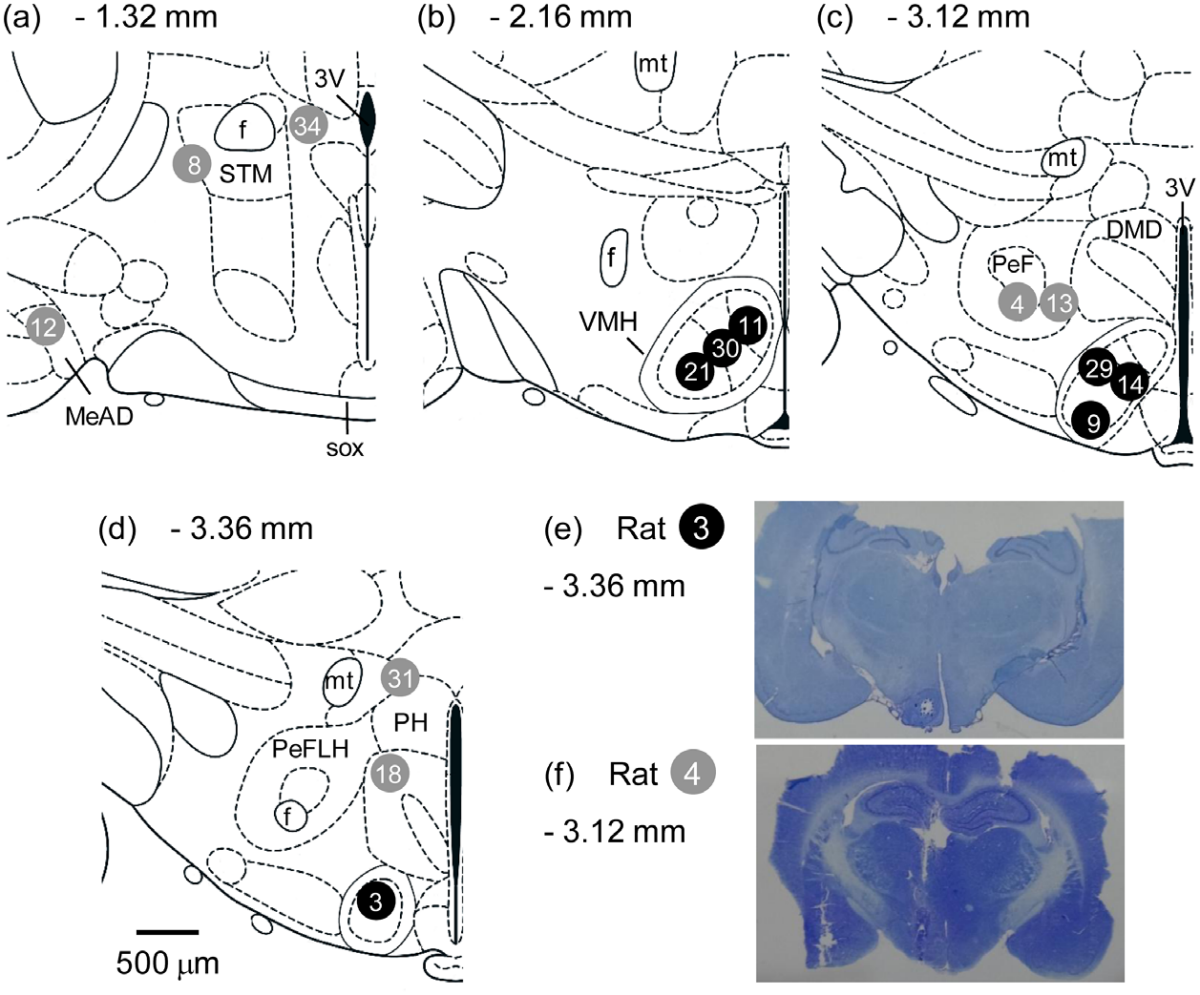
Coronal sections taken from the Paxinos and Watson atlas (2007) showing the location of the thermistor tips (panels A–D). The numbers above the schematic representation of each hypothalamic section indicate the distance (in mm) between the section plane and the bregma. Legends: 3V, 3rd ventricle; DMD, dorsomedial hypothalamic nucleus, dorsal part; f, fornix; MeAD, medial amygdaloid nucleus, anterodorsal part; mt, mammillothalamic tract; PeF, perifornical nucleus; PeFLH, perifornical part of lateral hypothalamus; PH, posterior hypothalamus; sox, supraoptic decussation; STM, bed nucleus of the stria terminalis, medial division; VMH, ventromedial hypothalamus nucleus. Photomicrographs of coronal sections (50 µm, cresyl violet staining) showing the location of the thermistor tips in two rats: one rat had a thermistor inserted into the frontal cortex, and the another animal had a thermistor inserted into the *caudate putamen nucleus* (panels E and F).

Figure 2 shows schematic drawings indicating the location of the thermistor tips defined by the deepest lesion observed in the brain tissue. These lesions are characterized by the absence of neural cells along the tract of the metal cannula or thermistor. In seven animals, the thermistor tips were observed in the VMH. However, in seven other rats, the thermistor was inserted in neural substrates that were lateral (rats #4, 8 and 12), dorsal (#8, 13, 18, 31 and 34) and rostral (#8, 12 and 34) to the VMH. Altogether, these substrates surrounding the VMH were termed the peri-VMH area.

**Figure 3:**
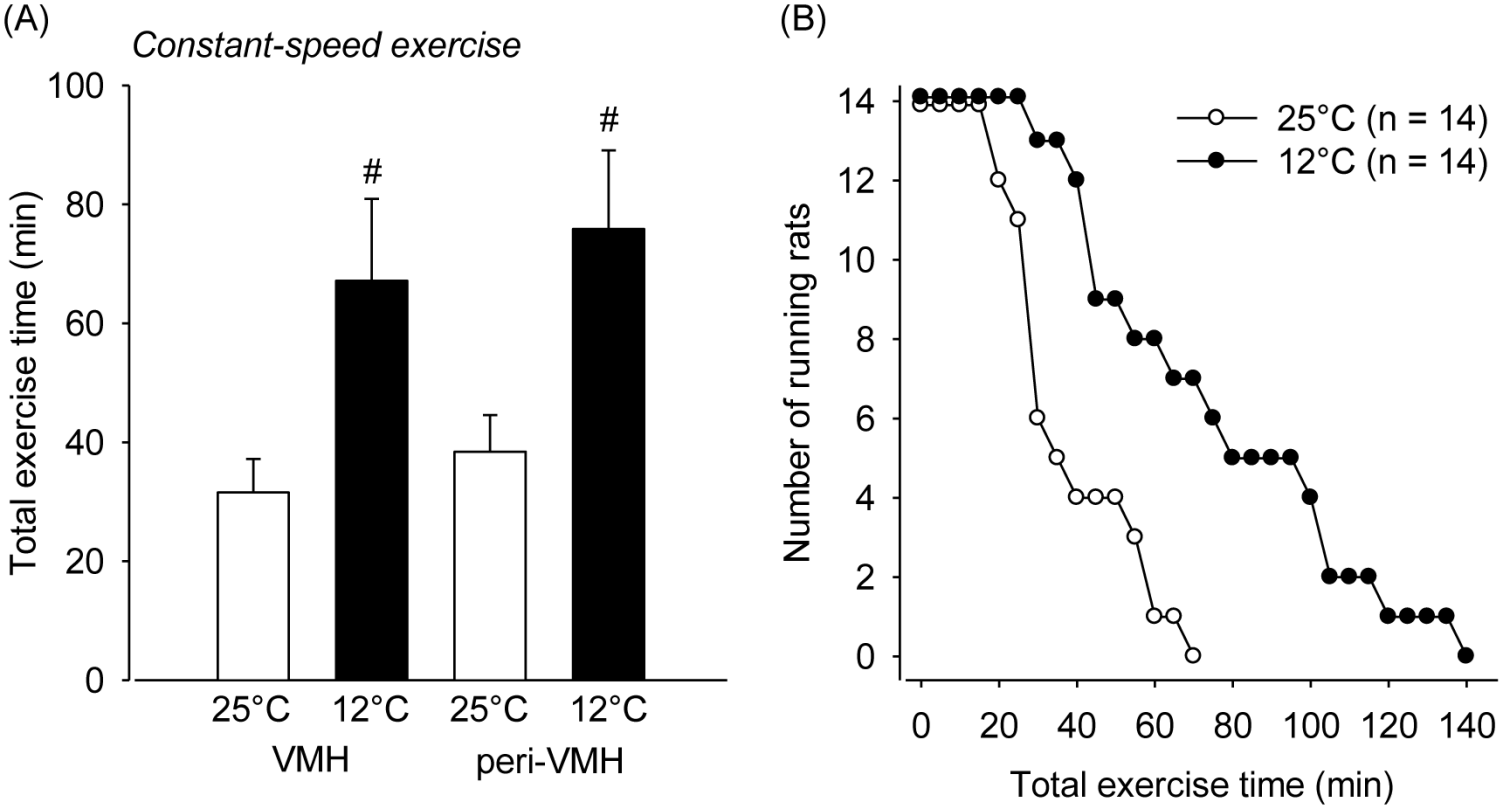
Total exercise time for the animals that were implanted with a guide cannula in the VMH or in the peri-VMH area. Constant-speed running (20 m/min and 5% inclination) was performed at ambient temperatures of 25°C and 12°C (panel A). #*P*<0.05 compared with the experimental trials at 25°C. The curves presented in the panel B show the maximum exercise duration tolerated by the rats subjected to the constant exercise sessions (panel B). The data are expressed as the number of rats that continued to run at specific time points at each ambient temperature.

The animals that were subjected to constant-speed treadmill running at 25°C ran for an average of 31.5±5.6 min (range: from 18 min to 55 min) until they voluntarily interrupted their effort. As observed for the incremental exercise, the TET was higher when the constant exercise was performed at the ambient temperature of 12°C than when the exercise was performed at 25°C (Figure 3A). This increased physical performance under cold conditions was observed both in the animals with thermistor tips inside the VMH (67.1±13.7 min vs. 31.5±5.6 min; *P* = 0.04; effect size = 1.28) and in animals with tips in the peri-VMH area (75.8±13.2 min vs. 38.4±6.1 min; *P* = 0.03; effect size = 1.37). Furthermore, regardless of the ambient temperature at which the exercise was performed, the TET of rats that underwent VMH temperature measurement was not different from the TET of rats that had underwent hypothalamic temperature measurement in areas near the VMH (*P* = 0.42 for 25°C and *P* = 0.65 for 12°C; Figure 3A). The logrank analysis also revealed the ergogenic effects associated with exercise under cold conditions. At the same time point at which all animals that exercised at 25°C had already fatigued, 50% (7 out 14) were still running at 12°C (*P* = 0.01; Figure 3B).

**Figure 4:**
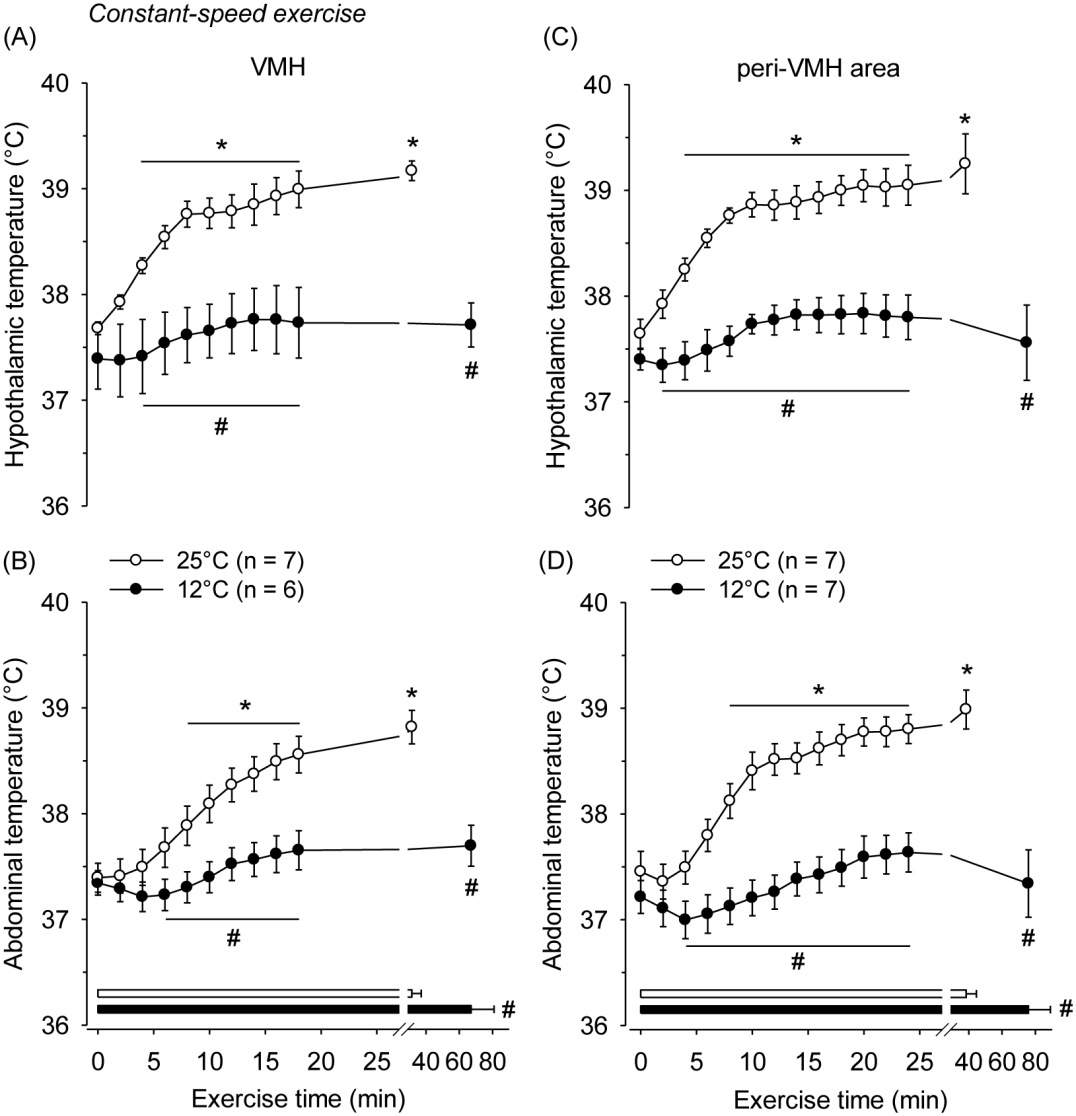
Body core temperature measured in the VMH (panel A), peri-VMH area (panel C) and abdomen (panels B and D) during constant-speed exercises that were performed until volitional fatigue at ambient temperatures of 25°C and 12°C. The horizontal bars indicate the total exercise time at 25°C (white bar) and 12°C (black bar). *P<0.05 compared with the beginning of exercise at 25°C; #*P*<0.05 compared with the experimental trial at 25°C.

During exercise at 25°C, the VMH temperature increased from the 4^th^ min, continued increasing until the 10^th^ min, and then reached a plateau that was sustained until volitional fatigue (39.16±0.09°C at fatigue vs. 37.68±0.06°C at the beginning of exercise; *P*<0.001; Figure 4A). In contrast, during exercise at 12°C, the VMH temperature remained unchanged throughout the running period (37.71±0.20°C at fatigue vs. 37.39±0.28°C at the beginning of exercise; *P* = 0.63). Moreover, analysis of the effects of ambient temperature revealed that the VMH temperature was lower from the 4^th^ min until the end of running at 12°C than at 25°C (Figure 4A). As expected, T_abd_ increased during the exercise at 25°C (38.81±0.15°C at volitional fatigue vs. 37.39±0.13°C at the beginning of exercise; *P*<0.001; Figure 4B). In the cold environment, T_abd_ remained unchanged throughout the exercise (37.69±0.19°C at fatigue vs. 37.34±0.11°C at the beginning of exercise; *P* = 0.20; Figure 4B).

The temperature measured in the peri-VMH area did not differ from the temperature measured inside the VMH at either of the studied ambient temperatures. At 25°C, the peri-VMH temperature increased from the 4^th^ min of running, continued to increase until the 10^th^ min and then reached a plateau, remaining elevated until volitional fatigue. In the cold environment of 12°C, the peri-VMH temperature remained unchanged throughout the fatiguing exercise. In addition, the peri-VMH temperature was lower from the 2^nd^ min until the end of running at 12°C than at 25°C (Figure 4C). As expected, the rats’ T_abd_ increased during the treadmill running at 25°C but was not changed at 12°C (Figure 4D).

**Figure 5:**
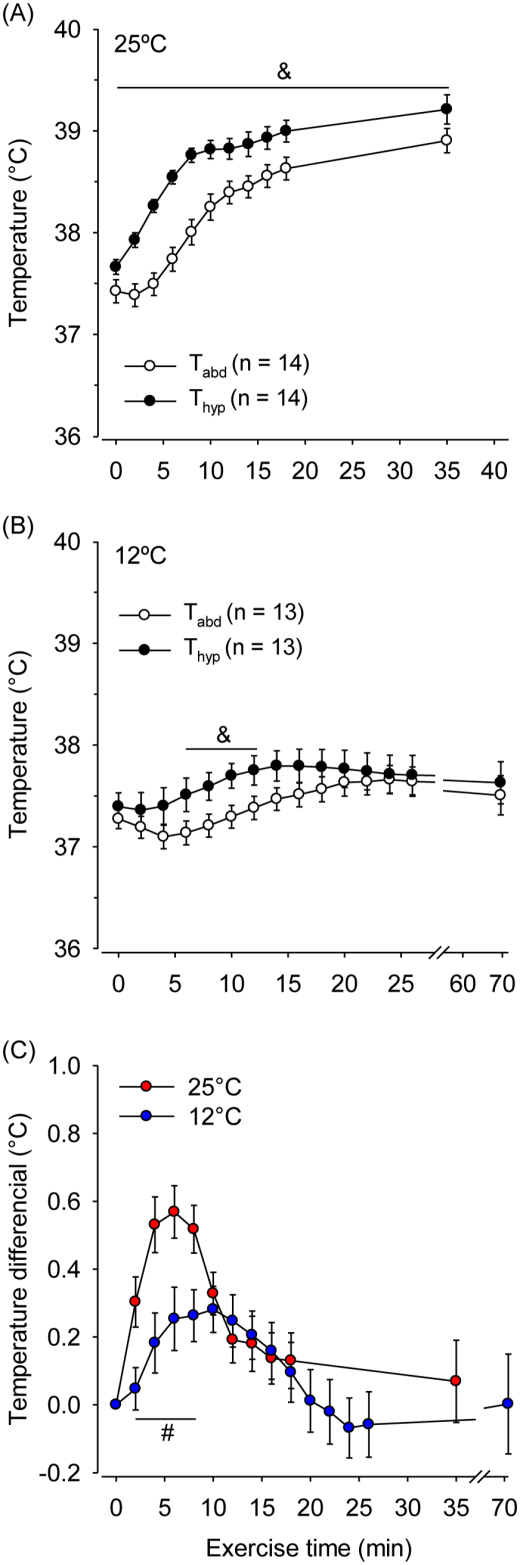
Hypothalamic and abdominal temperatures during constant-speed exercises that were performed until volitional fatigue at ambient temperatures of 25°C (panel A) and 12°C (panel B). Panel C shows the hypothalamic-abdominal temperature differentials at both ambient temperatures. &*P*<0.05 compared with the abdominal temperature; #*P*<0.05 compared with the experimental trial at 25°C.

The VMH and peri-VMH temperatures responded similarly to physical exercise at both temperatures; thus, we pooled the data from the animals that had temperatures measured at different sites of the hypothalamus and then compared the dynamics of exercise-induced changes in T_hyp_ and T_abd_ (Figure 5). T_hyp_ was higher compared with T_abd_ throughout the exercise period at 25°C (37.66±0.07°C vs. 37.42±0.11°C at the beginning of exercise; 39.20±0.14°C vs. 38.90±0.11 at volitional fatigue; *P* = 0.01 and 0.03, respectively). At 12°C, T_hyp_ was slightly but significantly higher compared with T_abd_ from the 6^th^ to the 12^th^ min of running (Figure 5B). The temperature differential increased during the initial min of running at 25°C, reached a peak at the 6^th^ min and then decreased toward pre-exercise values (Figure 5C). External cooling attenuated the increase in the temperature differential, which was observed at a single time point (i.e., the 10th min). Thus, the temperature differential was lower at 12°C relative to 25°C from the 2^nd^ to the 8^th^ min of running. Thereafter, no differences were observed in the temperature differential between the two ambient temperatures.

**Figure 6:**
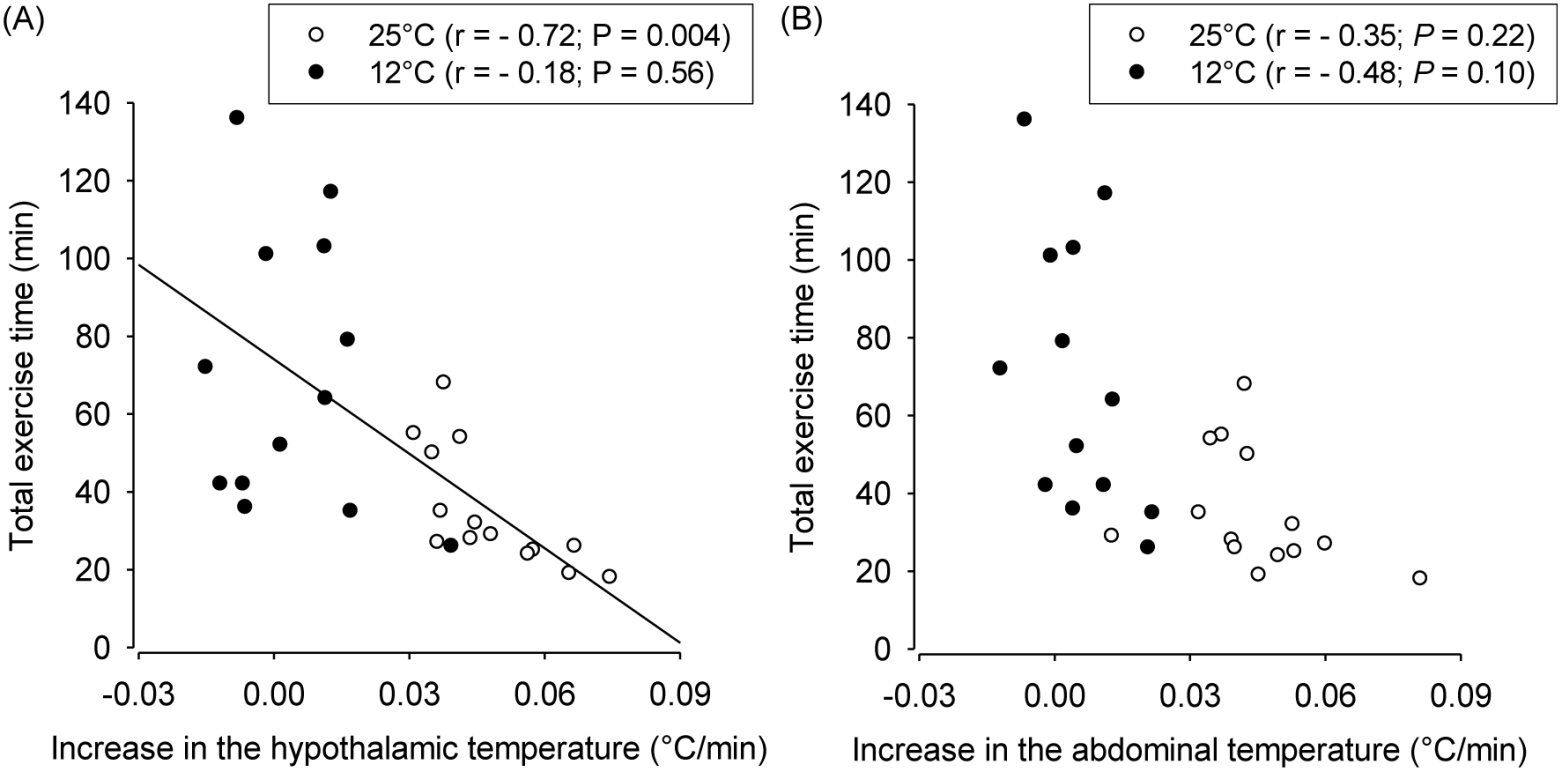
Correlation between the rate of increase in the hypothalamic temperature (panel A) or the abdominal temperature (panel B) and the total exercise time during constant-speed running at ambient temperatures of 25°C and 12°C.

Next, the association between the different indexes of T_core_ and physical performance was addressed. At 25°C, we observed a significant negative correlation between the rate of increase in T_hyp_ and the total exercise time (r = –0.72, *P* = 0.004). In contrast, no significant correlations were observed between the rate of increase in T_abd_ and physical performance during treadmill running at 25°C (r = –0.35, *P* = 0.22; Figure 6B). When the animals ran at 12°C, no significant correlations were observed between the running performance and the rate of increase in T_hyp_ (r = −0.18; *P* = 0.56; Figure 6A) or T_abd_ (r = –0.48; *P* = 0.10; Figure 6B).

## Discussion

The present study showed that the increase in T_hyp_ induced by fatiguing exercise at a constant speed of 20 m/min was prevented when rats were externally cooled by exposure to a cold environment. Moreover, under these experimental conditions, the physical performance was higher compared with the same exercise at 25°C, when the expected increases in T_hyp_ and T_abd_ were observed. This study also revealed the existence of different dynamics in the exercise-induced increases in T_hyp_ and T_abd_ at 25°C. Interestingly, these different dynamics were not observed when exercise was performed under external cooling conditions.

The running time until volitional fatigue was 97% to 113% higher during the constant-speed exercise performed at 12°C than during the exercise at 25°C (Figure 3). This result indicates that there is an optimal cold ambient temperature associated with the highest physical performance for a given exercise intensity, a finding that was previously described in exercising humans. Galloway and Maughan [34] analyzed the physical performance of subjects that cycled at 70% of their VO_2max_ at four different ambient temperatures and reported that the cycling time until volitional fatigue at 11°C was prolonged compared with that in all other experimental trials (4, 21 and 31°C).

The improved physical performance of our rats may be a consequence of the higher aerobic capacity observed in the cold because the maximum speed achieved during the incremental-speed exercise at 12°C was 18.8% higher than that at 25°C (Table 1). An immediate consequence of this finding is that the constant-speed running (20 m/min) was performed at ∼66% of the maximum speed attained at 12°C, whereas it was performed at ∼78% of the maximum speed at 25°C; therefore, the rats most likely ran more during the constant exercise in the cold because they expended less effort. Another hypothesis to explain the improved performance is that cold conditions attenuate exercise-induced hyperthermia [34], thus decreasing the perceived effort, which is intimately related to T_core_ [12], [35]. In support of this hypothesis, several lines of evidence show that cooling of the body core prior to [36] or during physical exercise [37] increases the subject’s endurance.

The lack of change in T_hyp_ and T_abd_ during constant treadmill running at 12°C (Figure 4) indicates that the rates of heat production and heat loss by the rat body were similar during this experiment. Physical exertion increases the metabolic rate of the body in an intensity-dependent manner; thus, exercise in a cold environment also possibly increases whole body heat loss, particularly via passive heat exchange processes [38]. An augmented heat loss in running rodents would be easily justifiable by the high ratio of body surface area to body mass [39] and by the experimental setup, which contained an electrical fan that circulated cold air and therefore enhanced the convection at the skin surface of the animals [1]. Furthermore, rats cannot properly engage in behavioral thermoeffectors aimed at heat conservation while they are running on a treadmill (e.g., they cannot assume a ball-like position).

The T_hyp_ changes measured in the VMH did not differ from the temperature changes measured in areas near the VMH (Figure 4) during the exercises performed at 25°C (when T_core_ increased) and 12°C (when T_core_ was unchanged). The absence of measured temperature differences at different hypothalamic sites suggests that the metabolic activation of VMH does not differ from the surrounding areas during exercise. In fact, several investigations have shown that changes in temperature of different brain areas occur mostly in parallel [18], [40].

At the beginning of constant-speed running at 25°C, T_hyp_ increased more rapidly compared with T_abd_ (Figure 5). Most likely, this early difference in the rate of increase of the T_core_ indexes resulted from intra-hypothalamic heat production rather than the delivery of warm peripheral blood, as indicated by the increase in the hypothalamic-abdomen temperature differential [18]. In addition, exercise promotes increased sympathetic outflow to the visceral vascular beds, which induces vasoconstriction and therefore allows a blood flow redistribution essential for the organism’s ability to meet the increased demands for oxygen and energetic substrates in the active muscles, lungs and brain [19]. The underperfusion of abdominal viscera (which is not highly active during exercise) may limit the delivery of the heat produced by the active organs and tissues at the beginning of exercise, thus slowing the rate of increase in T_abd_.

The faster increase in T_hyp_ compared with the increase in T_abd_, which was observed during the initial min of exercise at 25°C, was prevented by external cooling (Figure 5C). Two hypotheses could explain this unexpected observation: external cooling reduced hypothalamic neural activation or increased local brain heat loss. The first hypothesis seems unlikely, as several previous studies have reported genetic activation in hypothalamic neurons, including those located in the VMH and peri-VMH area, in response to cold stimuli [41], [42], [43]. The second hypothesis suggests locally augmented heat loss in the brain of running rats. Although the head is covered by a layer of fur, the airflow generated by the electrical fan may have removed the isolating air layer trapped in the fur and then facilitated convective heat loss near the head. Moreover, the heat loss from the brain is greatly dependent on local blood flow. As isolated stimuli, both exercise [7] and cold exposure [44] can increase cerebral blood flow; thus, we hypothesize that the combination of both factors would further increase the local blood flow and consequently increase brain heat loss during the initial min of running. As exercise continues, the lower temperature of the arterial blood that leaves the cold thorax and then perfuses the brain may also help prevent increases in brain temperature, contributing to the unchanged T_hyp_ during treadmill running at 12°C.

In the present study, T_abd_ and T_hyp_ were measured using telemetric devices and thermistors, respectively. This fact raises an important methodological issue because telemetric devices have high thermal inertia and may not accurately measure rapid changes in T_abd_ [13]. Therefore, the methodology employed here may have overestimated the faster increase in T_hyp_ that occurred in response to exercise initiation, but this methodology does not preclude comparisons between environments, as the temperature differential was calculated the same way at 12°C and 25°C. Additionally, our observations support the findings from research measuring abdominal aorta and brain temperatures with thermocouples in rats subjected to several arousing stimuli, which reported faster increases in brain temperatures compared with abdominal aorta temperatures [18].

As a side finding, during exercise at 25°C, we observed a significant negative correlation between the rate of T_hyp_ increase and the time until volitional fatigue but not between the rate of T_abd_ increase and the time until fatigue (Figure 6A and B). This finding suggests that T_hyp_ is a more sensitive signal than T_abd_ for determining physical performance, and this hypothesis is consistent with results previously reported by Caputa *et al.* [45], who showed that when the exercise was performed with a T_abd_ of 43°C and a T_hyp_ of 42°C, the animals managed to complete 60 min of exercise. However, when T_abd_ was maintained at 40°C and T_hyp_ was allowed to reach values higher than 42°C, the animals did not complete the exercise. Nevertheless, caution must be exercised before suggesting that T_hyp_ is a more sensitive signal for determining performance. First, correlation analysis does not determine a cause-and-effect relationship between two parameters. Second, interspecies differences exist, and the data collected in exercising goats may not be directly translated to exercising rats because rats (not treated with drugs) barely run with T_abd_ and T_hyp_ levels above 42°C [13], [14], [15].

In conclusion, the present findings indicate that T_hyp_ and T_abd_ are regulated by different mechanisms in running rats and that external cooling affects the relationship between both T_core_ indexes observed during exercise without environmental thermal stress. Our data also indicate that attenuated hypothalamic hyperthermia may contribute to improved performance in cold environments.
